# Irish Medical Association

**Published:** 1861-09

**Authors:** 


IRISH MEDICAL ASSOCIATION.—The annual meeting of this Association was held in the Royal College of Surgeons in Ireland, on Monday, the 3d of June. The chair was taken by Dr. Mackesy, of Waterford. The Report referred to the part taken by the Association in reference to the Bill for the Amendment of the Poor-law and Medical Charities Act, and for the registration of births, deaths, and marriages in Ireland. Early in October a subcommittee was appointed to make preparations for the approaching session of Parliament. The committee, after giving much time and labor to these subjects, prepared their report, the substance of which was: That the

Poor-law inquiries into charges against medical officers are investigated and conducted in a manner contrary to the spirit of the British Constitution; that the exclusion of the cases of injury and sickness from the Poor-house Hospital is oppressive to the poor and embarrassing to the medical officers; that the law and orders regulating vaccination are unsatisfactory and inefficient, and the remuneration quite inadequate to the importance of the duty performed; that the salaries of the medical officers are generally insufficient and disproportioned to the duties required of them; that a provision for granting pensions or superannuation allowances to medical officers is urgently needed and called for; and, finally, that the proportion of medical Inspectors to the Poor-law Inspectors should be rather increased than diminished, as proposed by the Poor-law Commissioners. Allusion was made to the refusal of the latter functionaries to sanction the increase voted by the Board of Guardians of the Abbeyleix Union, by a large majority, of the salaries of all their medical officers to £100 per annum, and to similar interference in other instances. Lastly, the deep regret of the Association was expressed for the severe losses it had sustained in the removal, by death, of some of its most distinguished members: Professor Porter, Drs. Robert C. Williams, Roe, Colvan, and Gore. Dr. Beatty was elected President for the ensuing year; Dr. Darby, Chairman of Council, and the Council was re-elected.—*Medical Times and Gazette.*

